# The role of social engagement in the association of self-reported hearing loss and health-related quality of life

**DOI:** 10.1186/s12877-020-01581-0

**Published:** 2020-05-25

**Authors:** Jiamin Gao, Hongwei Hu, Lan Yao

**Affiliations:** 1 Guanghua School of Management, Peking University, No. 5, Yiheyuan Road, Haidian District, Beijing, 100871 PR China; 2 School of Public Administration and Policy, The Research Center for Health Protection, Renmin University of China, No. 59, Zhongguancun Street, Haidian District, Beijing, 100872 PR China; 3 School of Public Administration and Policy, The Research Center for Health Protection, Renmin University of China, No. 59, Zhongguancun Street, Haidian District, Beijing, 100872 PR China

**Keywords:** Health-related quality of life, Hearing loss, Social engagement, Older adults

## Abstract

**Background:**

Hearing loss is highly prevalent and associated with reduced well-being in older adults. But little is known about the role of social factors in the association of hearing difficulty and its health consequences. This study aims to examine the association between self-reported hearing loss and health-related quality of life (HRQoL, consisted of physical and mental component summary, PCS and MCS), and to investigate whether social engagement mediates this association.

**Method:**

Data on 4035 older adults aged 60 years or above from a cross-sectional nationally representative database in China were obtained to address this study. HRQoL was measured by the Short Form 12 Health Survey (SF-12). Hearing loss was defined by a dichotomized measure of self-reported hearing difficulty, which has been proved to be sensitive and displayed moderate associations with audiometric assessment in elderly population. Social engagement was measured by the Index of Social Engagement Scale. Bootstrap test was applied to test for the significance of the mediating role of social engagement.

**Results:**

Self-reported hearing loss was found negatively associated with HRQoL in older adults, and hearing loss was much more related to reduced mental well-being. Social engagement played a partial mediating role in the association of hearing loss and HRQoL. Social engagement account for 4.14% of the variance in the change of PCS scores and 13.72% for MCS, respectively.

**Conclusion:**

The study lends support to the hypothesis that hearing loss is associated with aging well beings, and the use of hearing aid or proper social engagement intervention may improve the quality of life among the elderly.

## Background

Health-related quality of life (HRQoL), an important concept to describe subjective well-being, is defined as the quality of life that directly or indirectly related to health [1]. It is a comprehensive and multidimensional construct that reflects an individual’s physical health, psychological state, social relationships and emotional well-being [2, 3]. Impaired HRQoL predicts mortality and demands of health service utilization [4, 5]. A study of using SF-12 scale to measure the HRQoL of older adults aged 65 or above and found that individuals with lower physical component summary (PCS) or mental component summary (MCS) scores were in higher risk of death and hospitalization [6]. Personal and environmental contextual factors can impact individuals’ health-related quality of life. Evidences from China suggest that socio-demographic factors (e.g. marital quality and living arrangement), socio-economic status (SES), lifestyle, chronic health conditions and the place of residence may account for the decline in HRQoL [7–12]. Sensory impairment, such as cataract disease and age-related hearing loss, is negatively associated with the elderly HRQoL [13, 14].

Hearing loss, ranked as the third most prevalent chronic medical condition in older adults, only exceeded by arthritis and hypertension [15]. Approximately 11% older adults aged 60 or above in China were diagnosed with hearing impairment in China [16]. Audiometric hearing loss is associated with impaired quality of life [17, 18]. Auditory dysfunction can impair individual’s function of information exchange and have adverse impact on individual’s emotional, behavioral and cognitive reactions [19]. Communication breakdown and social isolation accompanied by hearing loss in older adulthood [20, 21] may result in anxiety, depression, impaired social interactions, physical dysfunctions [22, 23]. In addition, adults with hearing loss were more likely to be associated with lower individual and household socio-economic status [24, 25], making them more exposed to adverse life events, unhealthy lifestyles and stress perceptions, which exceed their coping capacity [26], resulting in higher risk for physical and psychological stresses [27–29]. Therefore, these stressors interact with neuronal plasticity and the immune system, which could contribute to the decline in health-related quality of life [30, 31]. Moreover, since hearing loss and financial strain can be treated as chronic stressors to individual’s wellbeing [32], older adults suffering with hearing loss as well as living in low-income households may face with dual risks of adverse health outcomes.

According to Social network theory, social network may influence health by promoting social participation and social engagement [33]. Social engagement, as interactions with potential ties in real life, provides individuals with a coherent and consistent sense of role identity, companionship and sociability as well. As a reasonable measure of social connectedness, engagement in social activities may foster communication, increase social capital and aide in increasing social resources and material goods, in order to cope with the negative impacts that physical stressor exerts on well beings [34]. Persons with age-related hearing loss may have challenges communicating verbally in the presence of background noise [35]. As a consequence, social gatherings or participation in social activities may become difficult and less enjoyed. Although previous studies have shown that social isolation may be one of the hypothesized mechanisms for the association between hearing loss and health consequences [36], empirical evidences on social engagement as a partial mediator in the relationship between hearing loss and HRQoL are very limited.

In a society with a large number of poor aging population, it is of great significance to identify the impacts of age-related hearing deficits on health and to find out the potential social intervention approaches. Although there are growing evidences on the association between hearing loss and quality of life in western countries, few studies have been done on this issue in China [37], particularly among poor individuals who may suffer from accumulated deficits of physical and economic difficulties. Moreover, most of studies in China were conducted with clinical series or other convenience sample [37–39], which may result in sample bias or overestimate the impact of hearing loss on health. In this study, we used a nationally representative and population-based data from Survey on the Aged Population of Low-income Families in Urban/Rural China (2018), aiming to examine 1) the association between hearing loss (HL) and health-related quality of life (HRQoL) among Chinese older adults aged 60 or above and living in a low-income household, and 2) the mediating role of social engagement in the relation of HL and HRQoL. The findings of this study will fill gaps on this issue in mainland China and contribute to the existing studies by addressing some limitations.

## Methods

### Participants

We obtained data from Survey on the Aged Population of Low-income Families in Urban/Rural China (2018), which was conducted from July 1 to September 31, 2018. The survey was approved and sponsored by the Ministry of Civil Affairs of the People’s Republic of China and carried out by the Institute of Social Science Survey, Peking University. The purpose of this survey was to investigate the well beings, family conditions, physical and psychological health status, social support, social networks, and health service utilizations of older adults aged 60 years or above who live in low-income households. The results of this survey were considered as the scientific basis for the formulation and implementation of low-income household policies for national and local governments in China [40].

Multistage, stratified and random-cluster sampling was used in this survey, which covered 155 counties (districts) and 1800 communities (villages) in 28 provinces, autonomous regions, and municipalities in China. Low-income family in this survey was defined as family whose annual household income per capita is below 1.5 times the local minimum living standard. Every three or four targeted households were matched with one geographically nearby general household as the control group. Home visits were conducted by trained investigators. All participants consented to participate in the survey, and the initial sample consisted of 6042 participants. In this study, we restricted our analysis to 4035 older adults living in low-income households with completed information on the questions we are concerned.

### Measures

The outcome variable of this study was the summary score of individual’s health-related quality of life. HRQoL was measured by the Chinese version 12-item Short Form Health Survey (SF-12), which has been tested with satisfactory reliability and validity in measuring health status of Chinese elderly population [41]. SF-12 was a HRQoL instrument based on the 36-item Short-Form Health survey (SF-36) [42, 43]. HRQoL measured by SF-12 was consisted of physical health and mental health component summary scores (PCS and MCS), reflecting individual’s physical and mental health status respectively. PCS an MCS were scored with a mean of 50 and a SD of 10 in the general population [43]. The higher the score, the better health and functional status.

The independent variable in this study was whether an individual had a self-reported hearing loss. Participants were asked the following question to check their hearing status (with hearing aid): “Do you have any difficulty with your hearing? The answers were coded as: 1. very difficult; 2. have some difficulties in hearing; 3. can hear clearly.” In this study, hearing loss was defined by a response of self-perceived hearing difficulties. Self-reported hearing loss, being regarded as a subjective assessment of hearing functions limitation, was treated as a binary variable (yes vs. no) in this study. Self-assessed hearing loss among elderly population reflects hearing disability. The validation of self-reported hearing loss in health research have been examined in prior study, and the results demonstrated that the single question for hearing loss yielded reasonable sensitivity and was minimally affected by age and gender, which could be recommended for use in assessing the magnitude of health burden caused by age-related sensory impairment [44, 45].

Social engagement was selected as a putative mediator variable based on theoretical assumptions from the literature. In this study, social engagement is assessed by the Index of Social Engagement (ISE), which was composed of six dichotomous items: (a) at ease interacting with others, (b) at ease doing planned or structured activities, (c) at ease doing self-initiated activities, (d) establish own goals, (e) pursues involvement in life of facility, and (f) accepts invitations into most group activities. The ISE scores ranged from 0 (lowest) to 6 (highest), and the scale demonstrated internal consistency and good reliability [46]. A cutoff point 2 was applied, and poor social engagement was referred to ISE scores below 2 [47].

Covariates in this study included sociodemographic, socio-economic, health behavior and health conditions characteristics, which were potentially associated with HRQoL according to previous studies [7, 12]. These variables specifically embraced age (continuous), sex (male vs. female), marital status (with spouse vs. without spouse), residence (urban vs. rural), education (illiterate vs. literate), household income (logarithm (X + 1), continuous), current employment status (yes vs. no), smoking (non-smoker, ex-smoker, vs. current-smoker), drinking (non-drinker, ex-drinker vs. current-drinker), and number of chronic disease (0, 1, 2, vs. 3 or above).

### Statistical analysis

Descriptive analysis of the mean and distribution of the study variables were reported for the study population. Difference between the study variables and hearing status were assessed using Chi-square test for categorical variables (sex, marital status, residence, employment status, smoking, drinking and number of chronic diseases) and a t test for continuous variables (age, HRQoL, social engagement). A Linear regression was applied to identify the association of the presence of hearing loss with PCS and MCS HRQoL. Nonparametric bias-corrected case resampling bootstrap method, which was valid in testing a single-mediator model [48], was employed to estimate the indirect effect of social engagement. Observations in which data were missing for one or more variables of each model were not included in the estimations. A *P*-value less than 0.05 was considered statistically significant. The software STATA 13.0(Stata Corporation, College Station, TX, United States) for Windows was utilized for statistical analysis.

### Results

Table 1 presents the sample characteristics. Overall, the mean age of the sample was 69.26 years (SD = 6.56), with 2230 males (55.71%) and 2460 older adults (61.61%) lived without spouse. In the sample, 2508 (62.65%), 3043(76.02%) and 1061(26.51%) individuals were in urban area, literate and employed after retirement, respectively. 2305(57.58%) adults were non-smoker and 2449(61.18%) did not drink. Besides, there were 1859(46.44%) cases had more than 3 chronic diseases, and the average household income per capita was 9600 RMB (SD = 10,141.16).

**Table 1:** Sample characteristics by self-reported hearing loss, N (%) or Mean (S.D.) ^+^(Table 1 is at the “Results” part)

Characteristic	Total Sample (*n* = 4003)	With HL (*n* = 1524)	Without HL (*n* = 2479)	*p*-value
Health-related quality of life				
Physical component summary (PCS)	42.10(7.37)	40.91(0.18)	42.83(0.15)	< 0.001^a^
Mental component summary (MCS)	41.11(11.47)	39.41(0.28)	42.16(0.23)	< 0.001^a^
Hearing loss				–
With hearing loss	1524(38.07)	–	–	
Without hearing loss	2479(61.93)	–	–	
Social engagement scores	3.94(1.95)	3.69(0.05)	4.10(0.04)	< 0.001^a^
Age^+^	69.26(6.56)	70.42(0.18)	68.54(0.12)	< 0.001^a^
Sex				0.844^b^
Male	2230(55.71)	846(55.51)	1384(55.83)	
Female	1773(44.29)	678(44.49)	1095(44.17)	
Marital status				< 0.001^b^
With spouse	1533(38.39)	647(42.59)	886(35.81)	
Without spouse	2460(61.61)	872(57.41)	1588(64.19)	
Residence				0.005^b^
Urban	2508(62.65)	913(59.91)	1595(64.34)	
Rural	1495(37.35)	611(40.09)	884(35.66)	
Education				< 0.001^b^
Illiterate	960(23.98)	441(28.94)	519(20.94)	
Literate	3043(76.02)	1083(71.06)	1960(79.06)	
Household income per capita^+^	9600.45(10,141.16)	7939.31(226.11)	10,624.40(216.84)	< 0.001^a^
Current employment status				0.039^b^
Yes	1061(26.51)	376 (24.67)	685(27.63)	
No	2942(73.49)	1148(75.33)	1794(72.37)	
Smoking history				0.706^b^
Never	2305(57.58)	884(58.01)	1421(57.32)	
Past	571 (14.26)	222(14.57)	349(14.08)	
Current	1127(28.15)	418(27.43)	709(28.60)	
Drinking history				0.073^b^
Never	2449(61.18)	941(61.75)	1508(60.83)	
Past	551(13.76)	227(14.90)	324(13.07)	
Current	1003(25.06)	356(23.36)	647(26.10)	
Number of chronic disease				< 0.001^b^
0	420(10.49)	120(7.87)	300(12.10)	
1	824(20.58)	246(16.14)	578(23.32)	
2	900(22.48)	340(22.31)	560(22.59)	
≥3	1859(46.44)	818(44.00)	1041(56.00)	

^a^ Student’s test. ^b^χ^2^ test

The PCS scores of the sample was 42.10(SD = 7.37) and 41.11(SD = 11.47) for MCS scores, respectively. One thousand five hundred twenty-four participants (38.07%) were self-reported with hearing loss and the mean scores of ISE was 3.94(SD = 1.95).

Table 1 also presents the bivariate analysis by hearing loss status. Older adults with hearing loss had lower scores of PCS and MCS, with 40.91 (SD = 0.18) for PCS and 39.41 (SD = 0.28) for MCS among adults with HL compared with 42.83 (SD = 0.15) for PCS and 42.16(SD = 0.23) for MCS among those without HL, respectively. Furthermore, individuals with HL reported lower scores of social engagement, with average score of 3.69(SD = 0.05), while that of non-HL group was 4.10 (SD = 0.04).

The multivariate association between HL and HRQoL (PCS and MCS), as well as the mediating role of social engagement in this relationship are shown in Table 2. Model 1 shows that there was a significant correlation between older adults with self-perceived hearing loss and low scores on PCS and MCS of HRQoL (*p* < 0.001). Compared with those without hearing loss, older adults with hearing loss had 1.92-point lower PCS scores and 2.76-point lower MCS scores, respectively. After adjusting for socio-demographic, SES and health conditions in Model 2, the association between hearing loss and health-related quality of life (both PCS and MCS) remained significant (*p* < 0.001). Age, sex, education, household income per capita and the number of chronic diseases were associated with HRQoL. Female and illiteracy had higher risk in lower PCS and MCS. As household income per capita increased, older adults were more likely to report better HRQoL. Moreover, with the number of chronic diseases increasing, the HRQoL of individuas tend to be lower.

**Table 2:** Regression results. (Table 2 is at the “Results” part)

Variables	Model 1	Model 2	Model 3	Model 4	Model 5	Model 6	Model 7
	PCS	MCS	PCS	MCS	ISE	PCS	MCS
**Self-reported HL**	−1.92(0.24)^***^	−2.76(0.37)^***^	− 1.19(0.23)^***^	−2.09(0.37)^***^	−0.24(0.06)^***^	− 1.14(0.23)^***^	− 1.81(0.36)^***^
**Social engagement index**						0.20(0.06)^***^	1.14(0.09)^***^
**Controls**							
Age			−0.04(0.02)^*^	0.23(0.03)^***^	0.004(0.01)	−0.04(0.02)^*^	0.23(0.03)^***^
Sex (ref. = male)			−1.28(0.31)^***^	− 1.47(0.49)^***^	0.06(0.08)	− 1.29(0.31)^***^	− 1.54(0.48)^***^
Residence (ref. = urban)			0.05(0.26)	−0.26(0.40)	0.02(0.07)	0.04(0.26)	−0.29(0.39)
Marital status (ref. = with spouse)			−0.39(0.24)	0.17(0.38)	0.32(0.07)^***^	−0.45(0.24)	−0.20(0.37)
Education (ref. = literate)			−0.15 (0.28)	−1.01(0.43)^*^	−0.47(0.07)^***^	− 0.05(0.28)	−0.47(0.42)
Log of household income per capita			0.51(0.13)^***^	1.73(0.21)^***^	0.23(0.04)^***^	0.47(0.13)^***^	1.46(0.20)^***^
Current employed (ref. = no)			1.14(0.27)^***^	0.34(0.42)	0.39(0.07)^***^	1.06(0.27)^***^	−0.11(0.42)
Smoking history (ref. = never)							
Past			−0.30(0.39)	0.03(0.61)	−0.08(0.11)	−0.28(0.39)	0.13(0.60)
Current			−0.12(0.32)	0.30(0.50)	−0.14(0.09)	−0.09(0.32)	0.46(0.49)
Drinking history (ref. = never)							
Past			0.37(0.38)	1.16(0.59)^*^	0.11(0.10)	0.35(0.38)	1.034(0.58)
Current			0.41(0.30)	1.92(0.48)^***^	0.38(0.08)^***^	0.34(0.30)	1.49(0.47)^***^
Number of chronic diseases			−1.89(0.11)^***^	−2.09(0.17)^***^	−0.16(0.03)^***^	−1.86(0.11)^***^	−1.91(0.17)^***^
**Constant**	42.83(0.15) ^***^	42.16(0.23)^***^	45.26(1.78)^***^	15.22(2.79)^***^	1.75(0.48)^***^	44.91(1.78)^***^	13.22(2.74)^***^
R^2^	0.0157	0.0134	0.1146	0.1052	0.0661	0.1171	0.1399

^*^p < 0.05, ^***^p < 0.001

Bootstrap for PCS: indirect effectβ = −0.049(0.019) ^**^,95% CI = [− 0.086,-0.012];direct effectβ = −1.136(0.223) ^***^, 95%CI = [− 1.574,-0.699]; total effectβ = − 1.185(0.028), 95%CI = [− 1.645,-0.644]

Bootstrap for MCS: indirect effectβ = − 0.277(0.076)^***^,95% CI = [− 0.427,-0.128]; direct effectβ = − 1.811(0.028) ^***^,95%CI = [− 2.624,-0.998]; total effectβ = − 2.088(0.392),95%CI = [− 2.756,-1.271]

Bootstrap 95%CI *P* < 0.05

Two OLS regressions were used to identify the mediating role of social engagement in the association between hearing loss and HRQoL. In Model 3, we used social engagement as a dependent variable and found that hearing loss was negatively associated with social engagement. In Model 4, after adjusting for social engagement and other covariates, hearing loss remained significantly associated with lower levels of PCS and MCS. Social engagement was related to HRQoL positively that social engagement raised in 1 score would be associated with 0.20-point increase in PCS and 1.14-points increase in MCS, indicating that social engagement played a meditating role in the linkage between HL and HRQoL.

**Fig. 1:**
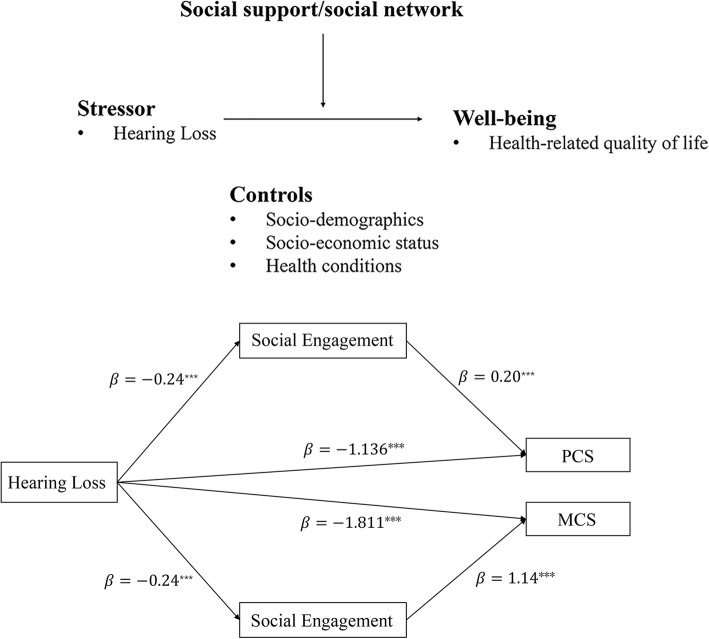
Coefficients for direct and indirect effects of self-reported hearing loss on physical and mental component summary on health related quality of life. * *p* < 0.05, ** *p* < 0.001, *** *p* < 0.005.

Figure 1 shows the coefficients for direct and indirect effects of self-reported hearing loss on PCS and MCS. Bia-corrected bootstrap procedure results further supported the mediation model. The indirect effects and 95% confidence intervals were − 0.049 [− 0.086,-0.012] for the partial mediating effects of social engagement on the association between hearing loss and PCS, demonstrating 4.14% of the variance in the change of PCS scores. Regarding to MCS, the indirect effects and 95% confidence intervals were − 0.277 [− 0.427,-0.128] for social engagement mediation, accounting for 13.27% of the variance in MCS scores. The bootstrap test result indicates statistically significant in both direct and indirect effect. Thus, social engagement mediates the linkage between hearing loss and HRQoL.

## Discussion

In the context of aging society with increased risk of chronic illness and a national policy implementation of poverty elimination, physical stressor’s impacts on well beings of individuals facing dual burden of aging and poverty have become growingly important social and health concerns. In this study, we conceptualized hearing loss as a chronic physical stressor, and estimated whether it was associated with health-related quality of life in a sample of older adults with poverty, utilizing a nationally representative survey data from China. Based on social network theoretical framework, we further tested whether social engagement played a mediating role in the relationship between HL and HRQoL.

First, our findings indicate that hearing loss was negatively associated with health-related quality of life among older adults with poverty in China. The impact of hearing loss on PCS and MCS remained statistically significant after controlling socio-demographic, SES and health related conditions. Our results comported well with previous studies which were relied on both standardized audiometric measurement techniques and self-perceived hearing loss, that impairment of hearing acuities was correlated to decreased scores on quality of life [18, 49]. Acquired hearing loss in old age is initially gradual and unpreventable [50]. Unaddressed hearing loss may give rise to physical dysfunction and cognition decline [51], further leading to worse self-perceived physiological quality of life. In addition, a study with a sample of older Australians suggested that hearing loss was predictive of poorer performance in social functioning and role limitation [17]. Furthermore, hearing loss was associated with communication breakdown, which may require long-term management of behavioral changes, and may suffer from loss of images, relationships and personal identity, resulting in anxiety, depression and other adverse psychosocial consequences [52].

Second, our results suggest that social engagement played a mediating role in the association of hearing loss and PCS and MCS. This result indicated that older adult’s social interactions with friends and family mediated the linkage between hearing loss, a common sensory impairment in older adults, and their physiological or psychological health [53, 54]. Social engagement or social connectedness, which may increase emotional exchange and companionship, have consistently been associated with health-related quality of life [55, 56]. Social exchanges or interactions can reduce feelings of being isolated or abandoned [57, 58].

Older adults with hearing loss may have difficulties in communication due to decline in hearing sensitivity [21]. Hearing loss may restrict individuals with effectiveness in social communication and interpersonal interactions, which limits one’s ability in building positive relationship with family and friends. Inactive engagement with life may predict lower life satisfaction [59], reduced quality of life [60] and increased mortality [61].

Meanwhile, there are also some limitations in this study. Firstly, our survey relied on self-reported hearing impairment with hearing aids, and the validity of these complaints could not be confirmed. Estimates based on self-reported sensory deprivation may be inaccurate given the tendency of older individuals to deny or underestimate their hearing difficulty. Secondly, since the medical history for auditory function as well as the severity of hearing loss were not known, we are not able to measure the impact’s duration of hearing loss exerting on HRQoL. Additionally, the causal relationship between hearing loss and HRQoL could not be verified, in that the descriptive, correlational, cross-sectional design was employed in this study.

Despite these limitations, the strengths of this study are also evident, including not only using a nationally representative population-based data in China to evaluate the association between self-reported hearing loss and HRQoL in older adults with poverty, but also identifying the role of social engagement in mediating the relationship. To our best knowledge, this is the first study to analyze the association between hearing loss and quality of life, along with the role of social engagement as a mediator among the older adults with poverty in China. Findings of this study have public health implications for Chinese policy makers to consider the importance of audiologic rehabilitation programs and the role of social support or social engagement in promoting quality of life among the elderly.

## Conclusion

Hearing loss is negatively associated with physical and mental health related quality of life among older adults with poverty in China. Social engagement mediates the linkage between hearing loss and HRQoL. In accordance with the stress-buffering and social network theory, hearing deficit, as a chronic physical stressor, can lead to poorer well-being, while social interactions could buffer (mediate) this relationship. Improvement in auditory function and social participation may contribute to promoting quality of life. Further studies are warranted to confirm our findings and to reveal the mechanism of hearing loss affecting quality of life, which will help identify the priority of hearing and health interventions for older adults.

## Data Availability

The datasets generated and/or analyzed during the current study are not publicly available due to issues of individual privacy, but are available from the corresponding author on reasonable request.

## Abbreviations

HRQoL: Health-related quality of life
PCS: Physical component summary
MCS: Mental component summary
SES: Socio-economic status
HL: Hearing loss
SF-12: 12-item Short Form Health Survey
SF-36: 36-item Short-Form Health survey
ISE: Index of Social Engagement
